# Low-Cost Predictors for Liver Function and Clinical Outcomes after Sustained Virological Response in Patients with HCV-Related Cirrhosis and Thrombocytopenia

**DOI:** 10.3390/medicina59010146

**Published:** 2023-01-11

**Authors:** Secil Omer, Adrian Iftime, Ileana Constantinescu, Ion Dina

**Affiliations:** 1 Department of Medical Semiology, Saint Joan Hospital Bucharest, Carol Davila University of Medicine, 042122 Bucharest, Romania; 2 Department of Biophysics, Carol Davila University of Medicine, 050474 Bucharest, Romania; 3 Department of Immunology and Transplant Immunology, Fundeni Clinical Institute Bucharest, Carol Davila University of Medicine, 022328 Bucharest, Romania

**Keywords:** hepatitis C virus, cirrhosis, thrombocytopenia, hepatocellular carcinoma, ascites, gastrointestinal bleeding, predictor, FIB-4, MELD, AFP

## Abstract

*Background and Objectives*: To find low-cost markers that can identify the hepatitis C virus cirrhotic patients that are at risk for long-term severe adverse liver effects (ascites, ascites or upper gastrointestinal bleeding, hepatocellular carcinoma), after treatment. There is established evidence for the benefits of treating hepatitis C virus cirrhotic patients, but there is still some need for clarification concerning the real impact on the long-term evolution after achieving sustained virological response; there is no general consensus in the literature about identifying the patients that do not improve post-treatment. *Materials and Methods*: Our retrospective analysis investigated the long-term (2 years) evolution of 46 patients with cirrhosis with thrombocytopenia, previously infected with VHC, treated and who obtained an SVR after DAA treatment. *Results*: Despite the overall improvement, 8.7% patients developed hepatocellular carcinoma and 6.5% patients ascites/upper GI bleeding. We found that FIB-4, MELD and AFP changes at 1 year were the most significant predictors for these outcomes. Additionally, a drop in leukocyte count after 1 year seemed to indicate a risk for hepatocellular carcinoma, but this was not consistent. *Conclusions*: It might be beneficial to intensify the surveillance for post-treatment adverse liver effects for the patients with these marker changes at 1 year.

## 1. Introduction

It is estimated that 71 million people worldwide are infected with the hepatitis C virus (HCV) [1] which can lead to advanced hepatic fibrosis, further related to disease complications such as hepatic insufficiency, hepatocellular carcinoma, upper digestive haemorrhage, ascites, hepatic encephalopathy and even increased mortality risk. HCV is the major cause of hepatocellular carcinoma (HCC) and cirrhosis-related mortality in developed countries [2].The reason for differences in the susceptibility to disease progression among individual patients is still incompletely understood [3].

Thrombocytopenia (TCP) is a haematological condition known to occur in chronically infected HCV patients. TCP in HCV-induced liver disease is often multifactorial and difficult to treat. It can be induced by: (a) an increased destruction of platelets (antibodies against platelets and/or hypersplenism) or (b) a decreased production of platelets (virus-induced bone marrow suppression and/or a decreased production of thrombopoietin) [4]. Hypersplenism is a common complication in patients with chronic HCV, leading to decreases in platelet and haemoglobin levels, and it correlates with the severity of cirrhosis. There is overwhelming evidence that standard coagulation testing does not accurately assess bleeding or clotting risk in patients with cirrhosis [5].

The prevalence of TCP is relatively large (an average of 24% of the HCV patients). Some studies reported an even higher prevalence (up to 45%) so there may be a substantial number of HCV patients at risk of bleeding complications and reduced likelihood of successful HCV antiviral treatment [6].

In the DAA (direct acting antivirals) era, the sustained virological response (SVR) rates with different treatment schemes are over 90%. Most of the cured patients have an excellent long-term clinical prognosis, even the difficult-to-treat categories such as patients with advanced fibrosis or interferon nonresponders [7]. This is the reason why multiple studies have tried to discover if achieving an SVR is also associated with a regression of fibrosis, portal hypertension and a reduction of disease complications.

A Canadian–European study of 350 patients with an SVR after interferon therapy found a significantly reduced frequency of both fatal and nonfatal hepatic complications over a period of 2.1 years [8]. Another study which followed patients with an SVR for 8.4 years, showed a reduction of 94% in mortality due to hepatic causes [9]. However, despite achieving an SVR, patients with HCV infection and advanced fibrosis are still at risk of disease progression, decompensation of cirrhosis and oncogenesis [10,11,12,13]. Bearing this in mind, there is a need to define criteria for the identification of those patients who will develop hepatic decompensation or hepatocarcinoma. Some studies have shown that the presence of cirrhosis and portal hypertension before achieving an SVR are associated with a greater risk of disease progression [12,14].

Most studies have monitored the evolution of liver stiffness with both transient elastography [15,16,17,18] and fibrosis scores that have been validated in the past years. There are also studies that tracked certain clinical parameters such as the evolution of disease until decompensation, hepatocarcinoma or transplant [19,20,21].

The evidence for the benefits of treating this group of patients has started to accumulate from recent studies, but there is still some need for clarification concerning the long-term impact on the evolution after achieving an SVR. The question to be answered is to identify (as early as possible) the patients who, despite the initial improvement after the SVR, will develop severe complications (fibrosis progression with consecutive decompensation, bleeding and/or hepatocarcinoma) in the long term.

In the present longitudinal study, we aimed to computationally identify the markers that could potentially indicate which patients with compensated cirrhosis (Child–Pugh A class) and thrombocytopenia are at risk for long-term severe adverse liver effects after treatment, despite initial improvement. Ideally, these found predictive markers would be low-cost or routinely performed analyses along the usual monitoring of these patients, since these adverse liver events are rare and most patients generally improve after treatment.

## 2. Materials and Methods

**Figure 1:**
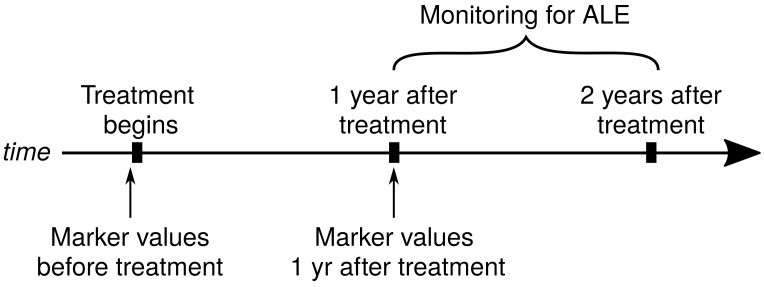
Timeline of the study.

We performed a retrospective longitudinal analysis on the evolution of patients from Saint Joan Hospital, Bucharest, diagnosed with HCV and treated, starting from 2019; the patients were still under our supervision at the time of writing the manuscript (see the timeline in Figure 1).

### 2.1. Inclusion Criteria

Patients with HCV infection—defined by positive anti-HCV antibodies and positive HCV–RNA (hepatitis C virus ribonucleic acid)—that were diagnosed with hepatic cirrhosis based on a fibrosis score of F4 with the Fibromax test (BioPredictive, Paris, France) and who had one of the following, oesophageal varices, thrombocytopenia under 150,000/µL or splenomegaly, were included.

SVR was defined by undetectable HCV–RNA, measured at 3 months after the end of therapy. All patients included in the study had an SVR. The method used for determining HCV-RNA was the COBAS AmpliPrep/COBAS TaqMan version 2 (lower limit of detection: 15 IU/mL) (Roche, Pleasanton, CA, USA) or the RealTime HCV assay (lower limit of quantification: 12 IU/mL) (Abbott Molecular, Des Plaines, IL, USA) according to the manufacturer’s instructions.

The DAA therapy used was sofosbuvir + ledipasvir for 15 of the patients and paritaprevir + ombitasvir + ritonavir + dasabuvir for the rest of the 31 patients. An SVR was achieved for all patients, regardless of the chosen therapy. The baseline Child–Pugh score was calculated within two months of the DAA treatment initiation.

### 2.2. Exclusion Criteria

Exclusion criteria were the presence of at least one hepatic nodule, prior hepatic decompensation (ascites, gastrointestinal haemorrhage, hepatic encephalopathy episodes) and the presence of haematological disease.

### 2.3. Variables Collected

We report the evolution of the following ten markers: total platelet count (nr/µL), leukocyte count (nr/µL), haemoglobin (g/dL), serum albumin (g/dL), serum glucose (mg/dL), alfa-fetoprotein (ng/mL), APRI score (AST to Platelet Ratio Index), MELD score (Model for End-Stage Liver Disease), CHILD score (Child–Pugh Score for Cirrhosis Mortality) and FIB-4 score (Fibrosis-4 Index for Liver Fibrosis).

The **APRI** score (aspartate aminotransferase to platelet ratio index) was calculated as: APRI = [(AST/upper limit of normal)/platelet count (10^9^/L)] × 100. The **FIB-4** score was calculated as: FIB-4 = age (years) × AST [U/L]/(platelet count (10^9^/L) × (ALT [U/L])^1/2^). The **MELD** score (Model for End-Stage Liver Disease) was calculated as: = 3.78 × ln[serum bilirubin (mg/dL)] + 11.2 × ln[INR] + 9.57 × ln[serum creatinine (mg/dL)] + 6.43. The **CHILD** score (Child–Turcotte–Pugh score as described by Pugh et al. [22]) was calculated using INR (international normalized ratio) values.

### 2.4. Timeline

We wanted to investigate the link between the appearance of adverse liver events and the dynamics in time of the above-listed markers. For this, we included in this study the marker values at these checkpoints:

- (a) Before treatment;
- (b) At the one-year follow-up visit;
- (c) For the dynamics of markers, we also calculated their changes in time (defined as: *the value at 1 year* − *value before treatment*), for each variable.

The adverse liver events (ALE) appeared after the 1st year post-treatment; we included in this study all the ALE that appeared in the period spanning between the 1st year after treatment and the 2nd year after treatment (see Figure 1); no ALE appeared before the 1st year after treatment in our group.

### 2.5. Software and Calculation

The raw data were gathered and stored in deidentified form in tabular sheets (Excel) from the hospital’s database. We performed the data analysis and visualization in *R* version 4.1 [23], with the additional packages *ggplot* [24], *tidyverse* [25], *logistf* [26].

For each variable, we calculated their descriptive statistics; we tested the normality of the distribution of variables using the Shapiro–Wilk normality test and by visually inspecting the quantile–quantile plots. If the distribution appeared to be normal, we compared the means of the groups using a paired *t*-test (Welch’s unequal variances *t*-test, which is a more robust version of Student’s *t*-test); otherwise, we used as the alternative the Wilcoxon signed-rank test.

We performed logistic regression tests using Firth’s bias reduction method, suitable for rare-occurrence events [27], using as predictors the variables listed above. We used this bias reduction method because ALE events are rare (in small proportion to the rest of the sample). Selection of the most likely predictor variable was done with bidirectional (forward and backward) stepwise multivariable logistic regression [28]. Briefly, for each situation, we started with the full model (incorporating all measured variables as the possible predictors) and then performed an automated stepwise regression eliminating redundant or low-impact variables from the models, thus selecting the most likely predictor variables. We used the AIC (Akaike information criterion) as a quality indicator for each step in the model selection; we selected the models with the lowest AIC scores and presented them in the Section 3. The overall significance of the logistic models was assessed with the likelihood-ratio test and Wald’s test. The significance level of all the tests used was set at the typical 5%.

The study followed the tenets of the declaration of Helsinki. The patient’s informed consent for treatment, monitoring and studying was recorded under the hospital’s guidelines.

## 3. Results

We followed up 46 patients with HCV infection, with an average age of 62.2 ± 8.9 years, treated at Saint Joan Hospital, Bucharest, in 2019. Four patients had HBV coinfection. Reactivation of HBV was seen in only one of them, at 6 months after the SVR. During the long-term follow-up (period of 1 year after treatment up to 2 years after treatment), seven patients developed rare severe adverse liver events: hepatocellular carcinoma (four patients) and ascites/upper gastrointestinal bleeding (three patients). The descriptive statistics of the patients are presented in Table 1.

### 3.1. Overall Group Evolution

As expected, over the first year after the treatment, there was a significant improvement of biochemical markers at the group level; we briefly present this improvement of serum markers below.

### 3.1.1. Platelets and Leukocyte Count, Haemoglobin, Serum Albumin—Evolution in Time

**Figure 2:**
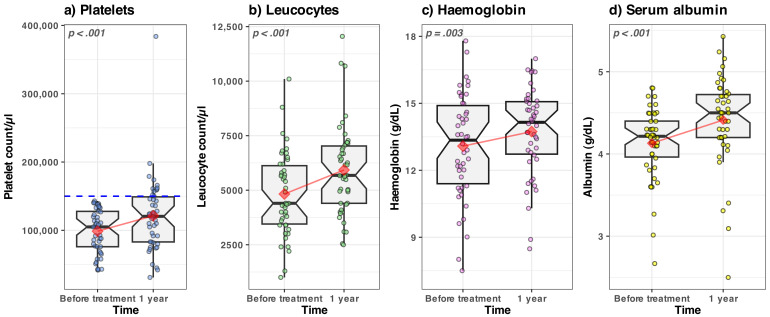
Boxplots of the serum markers evolution in time. The values recorded from each patient are represented by the dots (slightly jittered for clarity). The median value is represented by the horizontal thick black line. The lower and upper hinges correspond to the first and third quartiles (the interquartile range). The whiskers extend to 1.5 times the interquartile range; data points outside the whiskers are potential outlier values. The notches extend to the 95% confidence intervals for comparing the medians. The mean value of each subgroup is represented by the red diamond; the connecting red lines are meant as a guide to the eye to spot the trend. The dashed line in panel (**a**) represents the threshold value of 150,000 platelets/µL.

The average platelet count, leukocyte count, haemoglobin, serum albumin all had a statistically significant upward trend after 1 year (Figure 2); there was no difference in trends between sexes. A statistical overview of the above serum markers’ changes is presented in Appendix A.1 and Table A1.

### 3.1.2. APRI, MELD and FIB-4 Scores—Evolution in Time

**Figure 3:**
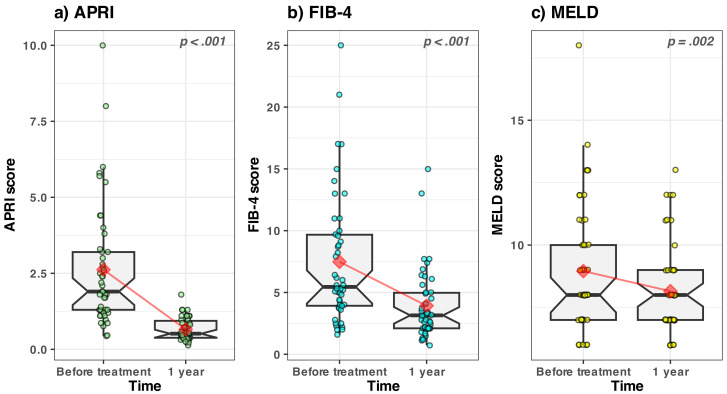
Boxplots of the APRI, MELD and FIB-4 score values vs. time. The values recorded from each patient are represented by the dots (slightly jittered for clarity). The median value is represented by the horizontal thick black line. The lower and upper hinges correspond to the first and third quartiles (the interquartile range). The whiskers extend to 1.5 times the interquartile range; data points outside the whiskers are potential outlier values. The notches extend to the 95% confidence intervals for comparing the medians. The mean value of each subgroup is represented by the red diamond; the connecting red lines are meant as a guide to the eye to spot the trend.

The APRI and FIB-4 scores were significantly improved after 1 year (Figure 3a,b). The median value of the MELD score was unchanged (i.e., 8.00, Figure 3c) even though the mean value slightly improved. The CHILD score was basically unchanged (and therefore not shown) with a median value 5.00, before and after the treatment. See Appendix A.2 and Table A2 for a statistical overview.

Based on the above presented results (Figure 2 and Figure 3), we can conclude that there was an overall objective improvement in the 1st year post-treatment in our group of patients.

### 3.2. Adverse Liver Events

Despite the biochemical improvement at the group level, during the long-term follow-up of the patients (interval between 1 year post-treatment and 2 years post-treatment), 8.7% developed hepatocellular carcinoma (HCC) and 6.5% ascites/upper GI bleeding (see Table 1).

We wondered if these severe rare clinical outcomes could be predicted from the simple inexpensive serum markers routinely collected and presented in the Section 2. We used logistic regressions as described in the Section 2, using all measured variables as predictors and as responses the following dichotomous outcomes:

1. The presence or absence of adverse liver effects (both HCC and ascites/GI bleeding);
2. Only the presence/absence of HCC;
3. Only the presence/absence of ascites/GI bleeding.

#### 3.2.1. Identified Concerning Factors for Adverse Liver Events (Both HCC and Ascites/Bleeding)

**Figure 4:**
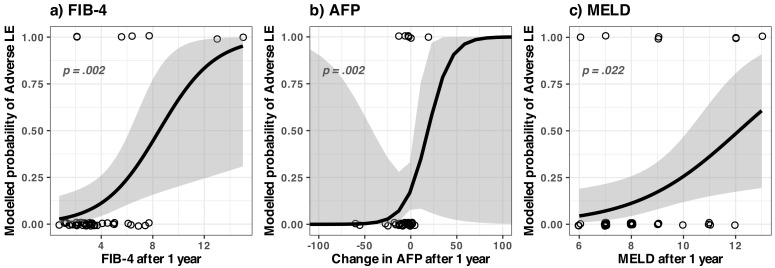
Modelled odds ratio (OR) of adverse liver events vs. nonevents as a function of FIB-4, AFP change and MELD scores. The grey bands represent the 95% confidence interval; only the statistical significant (*p* < 0.05) predictors are displayed; see Table A3 for the full numerical model.

In our dataset, the variables that were statistically significant linked to higher odds ratio of adverse liver events vs. nonevents were:

- FIB-4 score at 1 year (Figure 4a)
  For one unit increase in the FIB-4 score at 1 year the overall odds ratio (OR) of developing adverse liver events (vs. not developing) increased by a factor of 1.65;
- An AFP increase after 1 year (Figure 4b)
  The AFP change (as defined in Methods, Section 2.4) is the *AFP value at 1 year* − *AFP value at the beginning.* For a unit increase in the AFP change, the OR increased by a factor of 1.0;
- MELD score at 1 year (Figure 4c);
  For a unit increase in the MELD score, the OR increased by a factor of 2.26.

We note that the FIB-4 score seemed to be a stronger predictor (*p* = 0.0029) than the MELD score (*p* = 0.0223), see Table A3. Adding value to the model but not as significant were the serum albumin before the treatment and the platelet count after 1 year (see Table A3 for full model). As a critical overview of the results, we note that for AFP, the results were severely skewed by the presence of a single point with an exceptionally high value compared to the rest (795 ng/mL).

Based on these findings, we conclude that for this dataset, the most important predictors for overall adverse liver events were probably FIB-4 at 1 year and MELD at 1 year.

#### 3.2.2. Identified Concerning Factor for Hepatocellular Carcinoma

**Figure 5:**
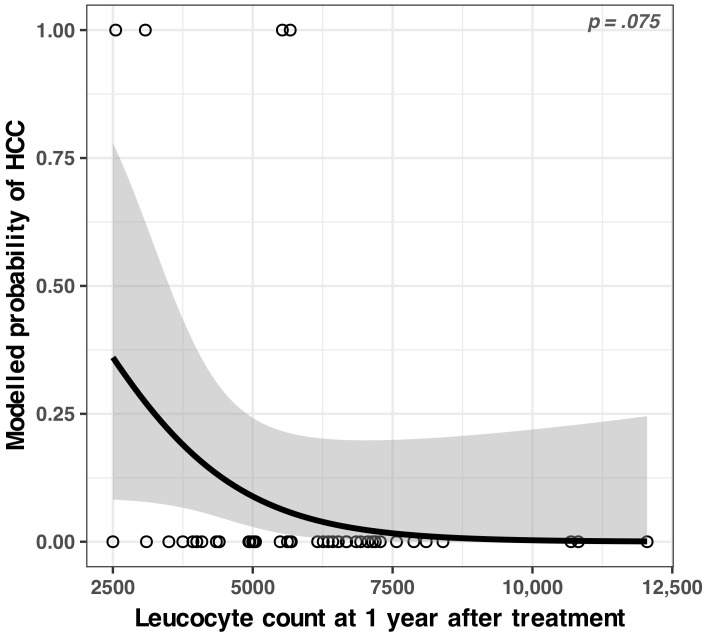
Modelled odds ratio (OR) of developing vs. not developing hepatocellular carcinoma as a function of leukocyte count after 1 year of treatment. The grey bands represent the 95% confidence interval; see Table A4 for the numerical model.

Using the same method as above, we did not find any significant predictor variable for hepatocellular carcinoma. Interestingly, the single variable that approached the significance threshold was the leukocyte count after 1 year of treatment. The model (see Figure 5 and Table A4) seemed to indicate that the risk of HCC could increase if the leukocyte count decreased, but this could not be asserted with statistical confidence.

#### 3.2.3. Identified Concerning Factor for Ascites/GI Bleeding

**Figure 6:**
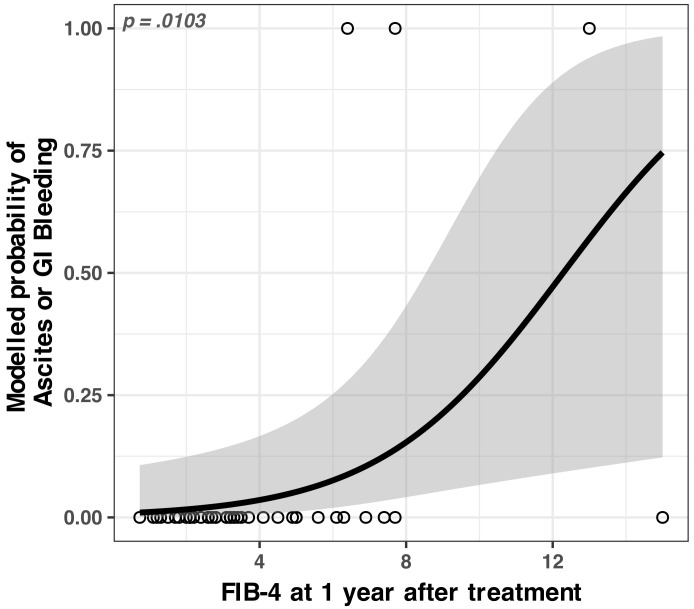
Modelled odds ratio (OR) of developing vs. not developing ascites/GI bleeding as a function of FIB-4 after 1 year of treatment. The grey bands represent the 95% confidence interval; see Table A5 for the full numerical model.

The FIB-4 score after 1 year of treatment was the single most important predictor for ascites/GI bleeding we found in our data set. For one unit increase in FIB-4 score measured at 1 year, the odds of developing ascites or GI bleeding (vs. not developing) increased by a factor of 1.4 (see Figure 6 and Table A5).

## 4. Discussion

### 4.1. General Improvement after Therapy

In the present study all patients obtained an SVR with DAA therapy and the biochemical results seemed to support the idea that overall liver function improved significantly.

#### 4.1.1. Haematological Improvements

Regarding the haematological alterations in cirrhosis type C, in our study we found a significant improvement of thrombocytopenia, considered as a marker of liver fibrosis and hypersplenism, at one year after the achievement of the SVR. The associated significant increase in leukocyte number (Figure 2b) is an important argument against the role of thrombopoietin synthesis normalization in the haematological changes associated with the SVR. The reduction of hypersplenism and inflammation, as well as the loss of viral (HCV) medullary inhibitory effect, are the most probable causes responsible for these haematological changes.

#### 4.1.2. Hepatic Function Improvements

As expected, after a year, the fibrosis scores FIB-4 and APRI had much improved after treatment in our patients, most probably a secondary reduction in inflammation.

In the study of Lu et al. [29], the authors also showed a significant reduction in both FIB-4 and APRI scores once the SVR was obtained. We cannot say for sure that these scores reflect a true reduction in hepatic fibrosis because these noninvasive markers can be overestimated by a high level of hepatic transaminases, meaning indirectly by inflammation [12,13,30]. In a study by D’ Ambrosio et al. [31], a significant reduction of noninvasive markers of fibrosis was also found, but serological tests had a suboptimal performance regarding the prediction of fibrosis level in patients with an SVR.

The MELD score calculated at one year after SVR also showed an improvement of liver function, proven also by a significant increase in albumin values at that time. MELD scores obtained in our group seemed to be in line with other studies that also found an improvement of the MELD score after the SVR, but also a stagnation for 17% and even a deterioration for 25% of patients [20,32,33].

### 4.2. Rare Adverse Liver Events

#### 4.2.1. Identified Concerning Factors for Adverse Liver Events (Both HCC and Ascites/Bleeding)

Despite the overall improvement at the group level, a minority of the patients developed severe adverse liver events in the period monitored (starting after the 1st year after treatment up to 2 years after treatment).

We found that in that subgroup, the FIB-4 and MELD scores at one year after the SVR were significantly correlated with the risk of adverse hepatic events. We conclude that these scores, measured one year after achieving the SVR, can potentially indicate a particular group of patients with a higher risk of adverse hepatic events. This subgroup of patients would mostly benefit from more frequent monitoring (as an overmonitoring of all patients might decrease compliance or can incur additional risks [34]).

In a previous study, these events were also correlated with pretreatment decompensation of liver disease, thrombocytopenia and hypoalbuminemia [35].

#### 4.2.2. Identified Concerning Factors for Hepatocellular Carcinoma

In our study group, four patients (8.7%) developed hepatic carcinoma in the second year after treatment. Concerning the incidence of hepatocarcinoma, previous studies have shown a correlation with the presence of cirrhosis, portal hypertension markers [36], or, differently, pretreatment hepatic disease decompensation and preSVR hypoalbuminemia [37,38]. In our dataset, we could not replicate the previously reported link between HCC and hypoalbuminemia [37]; we think that this might be due to different sample sizes between these studies and/or a difference in demographics.

*(a) Observations about the leukocyte count*

Among the various regression models, we tested in our dataset, the single variable that showed a stronger correlation with HCC was the leukocyte count at one year after the SVR. The lower the leukocytes count at one year after the SVR, the higher was the risk of developing hepatocarcinoma; however, as we stated in the Section 3, it did not pass the stringent significance tests defined for this study. Whether this result is a statistical fluke or a genuine link, only further studies can show. However, since this is an inexpensive, commonly performed laboratory analysis, we suggest that it might be beneficial for the patients to consider that a drop in leukocyte count after 1 year might warrant further attention to possible adverse liver events, including HCC.

*(b) Observations about FIB-4 and oncogenesis*

Previous research [39] regarding the risk of carcinogenesis after obtaining an SVR showed that, paradoxically, the risk of HCC increased with DAA-SVR, as opposed to the patients treated with interferon therapy. Later meta-analyses found [40,41] that the risk of hepatic carcinogenesis was the same for both patient groups—the ones treated with DAA and the one treated with interferon. Other studies claimed that obtaining an SVR was associated with a reduced incidence of HCC by 2.5–5 times, but a persisting risk for F3–F4 patients [42,43,44].

**Figure A1:**
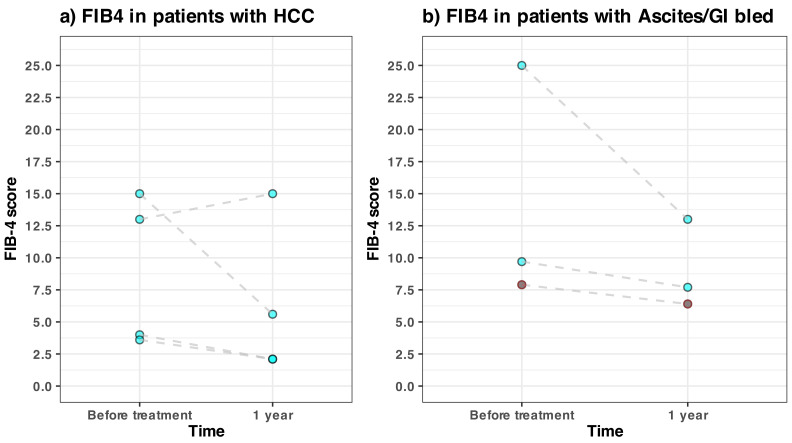
(**a**) The individual FIB-4 scores for patients with (**a**) hepatocellular carcinoma and (**b**) with ascites/GI bleeding, measured before and 1 year after the treatment. The brown dots in (**b**) represent the FIB-4 values of the patient that died during the 2nd year of follow-up.

Previous studies [45,46] found that the risk for HCC was very low in patients with FIB-4 under 3.25, a fact that was also confirmed by the patients in our study, who initially had an FIB-4 score of at least 3.6 (see Figure A1a for a detailed overview).

In another study [47], which involved patients who obtained an SVR with interferon therapy, the cut-off value of FIB-4 associated with a significantly lower rate of hepatocarcinoma was under 2.5 (*p* = 0.0003). However, this was not confirmed by our study; two of our patients who developed hepatocarcinoma had an FIB-4 score of 2.1 at 1 year after DAA therapy with an SVR (see Annex, Figure A1a). This raises questions regarding the safety of using the score FIB-4 for identifying the subgroup of patients with a higher risk of hepatocarcinoma after obtaining SVR. A multitude of studies have tried to explain the mechanisms behind oncogenesis after obtaining an SVR. Some of the mechanisms suggested, without reaching agreement, were a reduction of T cell reactivity to different epitopes associated with hepatocarcinoma [48], inactivation of certain interferon stimulated genes secondary to viral clearance [49] and also genetic and epigenetic alterations [50,51,52,53,54,55,56].

#### 4.2.3. Identified Concerning Factors for Ascites/GI Bleeding

Regarding the risk of liver disease decompensation, it is known that the viral clearance has been associated with a decrease in the frequency of these episodes (see for instance [19] where the authors reported a decrease from 18% in the first 6 months after antiviral therapy to 7% between 6 and 15 months after obtaining SVR).

In our group, a similar percentage of the patients (6.5%) developed decompensation episodes, which appeared in the second year after treatment. Amongst the monitored parameters in our study, the one with the highest predictability for decompensation was FIB-4 measured at 1 year. As stated earlier, in our study group, decompensation was seen in the second year of follow-up, and one of the patients concerned unfortunately passed away after a severe upper gastrointestinal bleeding episode. It is most likely that these patients were the ones where fibrogenesis continued despite a sustained viral response. All our patients who developed decompensation of the liver disease had an FIB-4 at 1 year of over 6.0 (see Figure A1 for a detailed overview).

Therefore, we suggest that the FIB-4 score measured at 1 year after treatment can potentially identify those patients that would benefit from a closer surveillance in order to prevent hepatic decompensation (ascites, GI bleed).

All the predictors that we found and discussed above (FIB-4 and MELD values, measured at 1 year after treatment, and the AFP increase after 1 year of treatment) are low-cost, routinely performed biochemical analyses, and our data suggest their usefulness in predicting severe adverse liver events. As a relative novelty, we would like to stress the fact that these are the values measured at 1 year after treatment; the past studies cited above focused mainly on the values measured at the beginning of the treatment. Further studies might indicate the strength of using the marker values at 1 year after treatment as predictors for the outcome in the second or later years past the SVR.

#### Limitations

We understand that our study has limitations; the variables we found as possible predictors for adverse liver events (hepatocellular carcinoma, ascites, upper GI bleeding) reflect only our limited dataset. As for any modelling done, we acknowledge that any model (including our models presented here) represent a simplification of the reality. We acknowledge the limits of our sample size: this was a pilot study. Platelet-to-lymphocyte and neutrophil-to-lymphocyte ratios are markers of systemic immune response that could be potentially used as prognostic for HCV infection severity [57], for HCC outcomes [58] and also for a range of other chronic hepatic diseases [59,60]. Dyslipidaemia [61,62] and insulin resistance [63] were also previously associated with the severity of HCV complications. We acknowledge that the present study did not analyse these potentially useful biomarkers, which could perhaps be incorporated in further studies. We strove to find the most influential predictors among the common low-cost serum markers, but we acknowledge the possible and very probable influence of other many confounding factors not included in this study.

## 5. Conclusions

Patients with Child–Pugh A hepatic cirrhosis and thrombocytopenia, previously infected with HCV generally ameliorate after DAA treatment; yet despite haematological and liver function improvement, a minority of the patients developed severe complications (hepatocellular carcinoma, ascites or upper GI bleeding), during the monitoring after 1 year post-treatment.

As the most important statistically significant predictors for all these severe outcomes, we found the FIB-4 and MELD values, measured at 1 year after treatment, and the AFP increase after 1 year of treatment. For hepatocellular carcinoma alone, we could not find a definite predictor, but the single most concerning factor found was a decrease in leukocyte count after 1 year of treatment (the risk of HCC decreased as leukocyte count increased).

## Figures and Tables

**Table 1:** Descriptive statistics of the patients. The adverse liver events were HCC (hepatocellular carcinoma), ascites and/or upper GI (gastrointestinal) bleeding.

Characteristic	Overall, N = 46 ^1^	Female, N = 29 ^1^	Male, N = 17 ^1^
Age (years)	64 (58, 67)	65 (61, 71)	57 (51, 66)
HCC	4 (8.7%)	3 (10%)	1 (5.9%)
Ascites, GI bleeding	3 (6.5%)	1 (3.4%)	2 (12%)

^1^ Median (IQR); n (%).

## Data Availability

The data presented in this study are available on request from the corresponding author. The data are not publicly available due to privacy and ethical restrictions.

## Abbreviations

The following abbreviations are used in this manuscript:

AIC	Akaike Information Criterion
APRI	AST to Platelet Ratio Index score
AST	Aspartate aminotransferase
CHILD	Child–Pugh Score for Cirrhosis Mortality
CI	Confidence interval
DAA	Direct-acting antivirals
FIB-4	Fibrosis-4 Index for Liver Fibrosis score
GI	Gastrointestinal
HBV	Hepatitis B virus
HCC	Hepatocellular carcinoma
HCV	Hepatitis C virus
HCV–RNA	Hepatitis C virus ribonucleic acid
INR	International normalized ratio
IQR	Interquartile range
MELD	Model for End-Stage Liver Disease score
OR	Odds Ratio
SVR	Sustained virological response
TCP	Thrombocytopenia

## Appendix A. Statistics

### Appendix A.1. Statistics of Serum Markers

For all the markers listed in Figure 2 and Table A1, we found a statistically significant difference between the values before the treatment and after 1 year:

- Average values of blood platelet count increased with an average of 22,600/µL (Wilcoxon signed-rank test with continuity correction, Z = 4.61, *p* < 0.001).
- The leukocyte count increased with an average of 1102/µL (Wilcoxon signed-rank test with continuity correction, Z = 4.31, *p* < 0.001).
- Haemoglobinemia increased with an average 0.65 mg/dL (Welch *t*-test, *t*(45) = 2.934, *p* < 0.003).
- Albuminemia increased with an average of 0.29 g/dL (Wilcoxon signed-rank test with continuity correction, Z = 4.09, *p* < 0.001).

**Table A1:** Serum markers values before the treatment and after one year.

Characteristic	Before Treatment, N = 46 ^1^	1 Year, N = 46 ^1^
Platelet count/µL	98,722 (32,220)	121,348 (55,928)
Leucocytes/µL	4826 (1889)	5928 (2055)
Hb mg/dL	13.08 (2.35)	13.73 (1.96)
Serum albumin g/dL	4.13 (0.44)	4.41 (0.55)

^1^ Mean (standard deviation).

### Appendix A.2. Statistics of Liver Scores

The numerical values of liver scores presented in Figure 3 are presented in Table A2. We found statistical significant changes between the values before the treatment and 1 year afterward for the APRI and FIB-4 scores:

- APRI had a significant decrease (Wilcoxon signed-rank test with continuity correction, Z = −5.81, *p* < 0.001);
- FIB-4 score had a significant decrease (Wilcoxon signed-rank test with continuity correction, Z = 5.56, *p* < 0.001).

The MELD score decreased slightly (Figure 3b), from an average value of 8.96 to 8.15, but the median value was unchanged (i.e., 8.00). The CHILD score was basically unchanged (i.e., 5.00) in this interval.

**Table A2:** Evolution of various liver scores before and after one year.

Characteristic	Before Treatment, N = 46 ^1^	1 Year, N = 46 ^1^
APRI Score	1.90 (1.30, 3.20)	0.52 (0.38, 0.94)
MELD Score	8.00 (7.00, 10.00)	8.00 (7.00, 9.00)
FIB-4 Score	5.4 (3.9, 9.7)	3.2 (2.1, 5.0)

^1^ Mean (first quartile, third quartile).

### Appendix A.3. Statistical Modelling

**Table A3:** Summary of the logistic model for rare events (Firth method, penalized method) presented in Figure 4. Likelihood ratio test = 20.70995 on 5 dfs, *p* = 0.0009, n = 46; Wald test = 10.2277 on 5 dfs, *p* = 0.069.

Characteristic	OR ^1^	95% CI ^1^	*p*-Value ^2^
(Intercept)	0.0000	0.0000, 0.0877	0.0212
FIB-4 at 1 year	1.6549	1.1814, 2.8482	0.0029
AFP change after 1 year	1.0077	1.0027, 1.0233	0.0025
MELD at 1 year	2.2616	1.1149, 6.8938	0.0223
Serum albumin, before treatment	3.3884	0.2711, 253.1818	0.3793 *
Platelet count at 1 year	1.0000	0.9999, 1.0000	0.0927 *

^1^ OR = odds ratio, CI = confidence interval; ^2^ Profile-penalized log-likelihood; ^*^ *p*-values higher than 0.05 were considered statistically nonsignificant.

**Table A4:** The summary of the model hepatocellular carcinoma (HCC) vs. leucocyte count at 1 year, presented in Figure 5. Logistic model for rare events (Firth method, penalized method). Likelihood ratio test = 3.1688 on 1 df, *p* = 0.0750, n = 46; Wald test = 2.7931 on 1 df, *p* = 0.0946.

Characteristic	OR ^1^	95% CI ^1^	*p*-Value ^2^
(Intercept)	2.3603	0.0747, 87.5143	0.6214 *
Leucocyte count at 1 year	0.9994	0.9985, 1.0000	0.07505 *

^1^ OR = odds ratio, CI = confidence interval; ^2^ Profile-penalized log-likelihood; ^*^ *p*-values higher than 0.05 were considered statistically nonsignificant.

**Table A5:** The summary of the model ascites and GI bleeding vs. FIB-4 at 1 year presented in Figure 6. Logistic model for rare events (Firth method, penalized method). Likelihood ratio test = 6.5676 on 1 df, *p* = 0.0103, n = 46, Wald test = 4.8434 on 1 df, *p* = 0.0277.

Characteristic	OR^1^	95% CI ^1^	*p*-Value ^2^
(Intercept)	0.0133	0.0007, 0.0863	<0.001
FIB-4 at 1 year	1.4049	1.0866, 1.9716	0.0103

^1^ OR = odds ratio, CI = confidence interval; ^2^ Profile-penalized log-likelihood.
